# Random neuronal ensembles can inherently do context dependent coarse conjunctive encoding of input stimulus without any specific training

**DOI:** 10.1038/s41598-018-19462-3

**Published:** 2018-01-23

**Authors:** Jude Baby George, Grace Mathew Abraham, Zubin Rashid, Bharadwaj Amrutur, Sujit Kumar Sikdar

**Affiliations:** 1 Center for Nanosicence and Engineering, IISc Bangalore, Bengaluru, Karnataka India; 2 Robert Bosch Center for Cyber-Physical Systems and Department of Electrical Communications Engineering, IISc Bangalore, Bengaluru, Karnataka India; 3 Molecular Biophysics Unit, IISc Bangalore, Bengaluru, Karnataka India

## Abstract

Conjunctive encoding of inputs has been hypothesized to be a key feature in the computational capabilities of the brain. This has been inferred based on behavioral studies and electrophysiological recording from animals. In this report, we show that random neuronal ensembles grown on multi-electrode array perform a coarse-conjunctive encoding for a sequence of inputs with the first input setting the context. Such an encoding scheme creates similar yet unique population codes at the output of the ensemble, for related input sequences, which can then be decoded via a simple perceptron and hence a single STDP neuron layer. The random neuronal ensembles allow for pattern generalization and novel sequence classification without needing any specific learning or training of the ensemble. Such a representation of the inputs as population codes of neuronal ensemble outputs, has inherent redundancy and is suitable for further decoding via even probabilistic/random connections to subsequent neuronal layers. We reproduce this behavior in a mathematical model to show that a random neuronal network with a mix of excitatory and inhibitory neurons and sufficient connectivity creates similar coarse-conjunctive encoding of input sequences.

## Introduction

**Figure 1:**
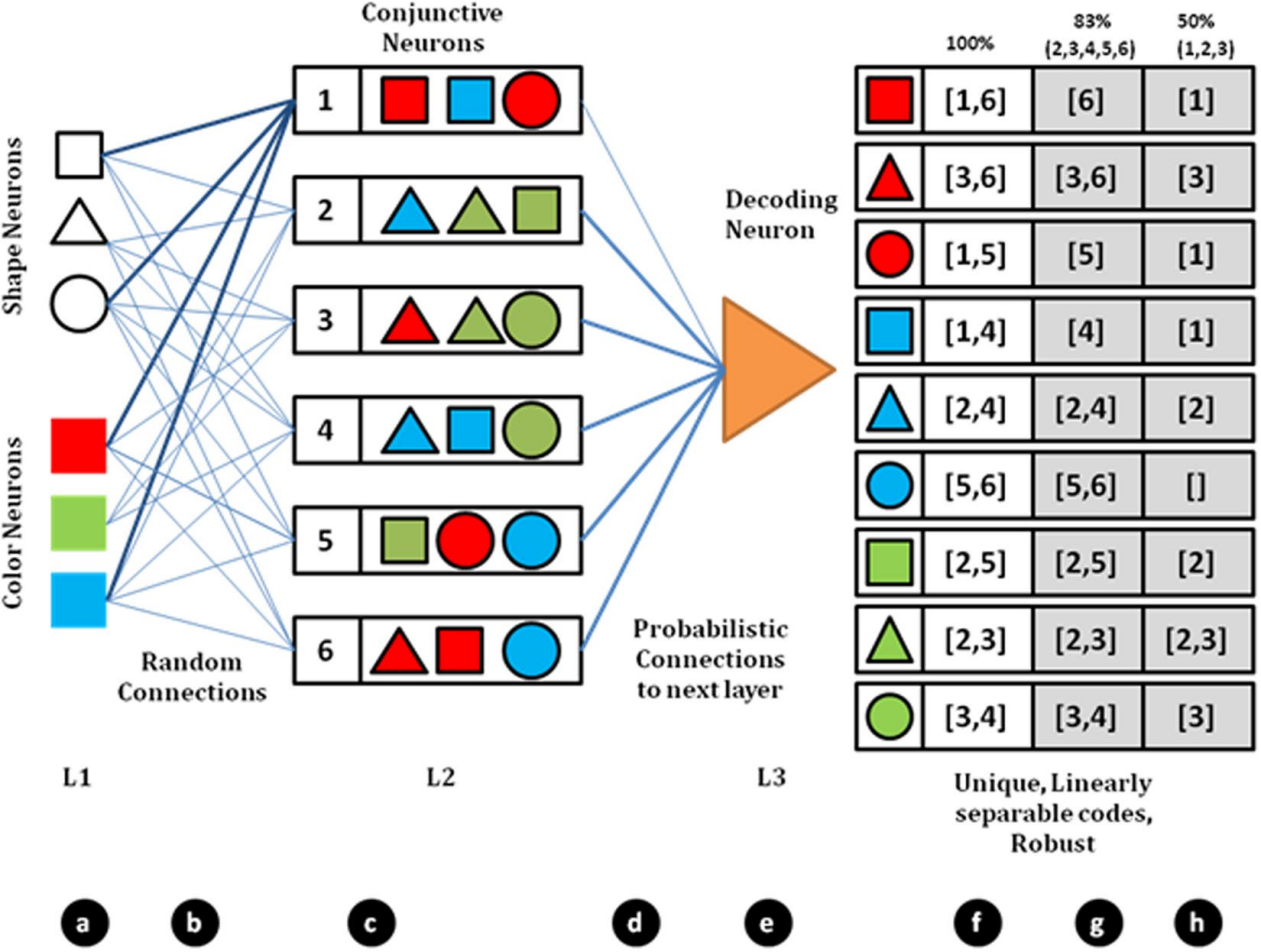
Architecture for pattern classification presented in this report. (**a**) L1 represents a set of input neurons. (**b**) These are connected to the next layer L2 probabilistically. (**c**) Such probabilistic connections give rise to coarse conjunctive neurons. As an example, Neuron 1 in L2 receives inputs from neurons coding for square, circle, red and blue and activates for the cases red square, blue square and red circle. With several such neurons in L2, a population code is formed. This is highlighted in f. When a red square is presented, neurons 1 and 6 are activated (say population code [1,6]) while for a blue circle, the population code is [5,6]. These codes are linearly separable (when considered as a binary vector in 6 dimensions). With such linearly separable codes, a single neuron in layer 3 (we have a perceptron as a proxy) can learn to decode any one of the unique population code using STDP mechanism. Even if the connection between layer 2 and layer 3 is probabilistic as in d, the code as seen by the perceptron is unique and linearly separable. For example, suppose a neuron in L3 does not receive a connection from Neuron 1 in L2, still the population code as seen by it (as shown in g) is unique for each pattern and it can decode the pattern. With further reduction in probability of connection (50%) as in h, the population code is no longer unique.

Pattern or sequence recognition and classification is a well-studied problem in engineering that uses biologically inspired architectures like artificial neural networks, and more recently deep learning networks that have shown promising results in solving such tasks. However, the learning algorithms adopted by these architectures require multiple iterations and modifications of the connectivity weights across all layers of the network. The existence of similar multi-layered learning in the biological neuronal networks for efficient processing of input stimuli and classification of inputs has not been observed yet experimentally. An alternative learning architecture is to have a random neuronal ensemble with a mix of inhibitory and excitatory neurons that is then connected to another layer of perceptron type neurons, in a probabilistic manner, with learning restricted to the final perceptron layer. We describe this further in the schematic in Fig. 1, where a layered neuronal system with probabilistic connectivity at input and output of first layer, is connected to a second layer having neurons equipped with STDP, to solve the problem of input classification without any need for network modification/learning at the input layer. We experimentally validate this architecture by using neuronal ensembles cultured on a multi electrode array, to form the first layer of the Fig. 1. The multi-electrode array allows us to create complex spatio-temporal input stimulation patterns, that get encoded by the neuronal tissue which is then observed as responses at the electrodes for further analysis. We show through modeling and by fitting experimental data that probabilistic connections and a layered architecture as in Fig. 1, can provide a very robust platform to implement context dependent classification. Our data and results show the presence and usefulness of coarse-conjunctive tuning of neurons in the random ensemble, as observed in the brain for pattern recognition. These results also suggest the usefulness of Liquid State Machines as a framework for brain-computations^1^.

The architecture shown in Fig. 1 mimics the ability of the brain to combine (or bind) multiple features of a stimulus into single percepts (the ‘binding problem’). This is illustrated by single neurons coding for 3 different shapes (square, triangle and circle) and 3 different colors in the first layer (L1). If we now consider an object with two features having both color and shape information for e.g. a red square, the brain does not perceive these features separately but as a composite red square. The brain solves this problem by having neurons that encode shape and color information conjunctively (the conjunctive neurons). This type of encoding is called ‘conjunctive encoding’. If we consider all possible combinations between color and shape, the combinatorial problem is huge and would require far too many neurons to adequately represent all possible conjunctions. The problem can be partially solved by having a coding mechanism with distributed representations where each neuron encodes a mixture of input features that become active at different levels to different combinations of the two features viz. color and shape. Thus, a single neuron instead of encoding for a single conjunction, codes for multiple conjunctions, i.e., the neuron does coarse conjunctive encoding. This is illustrated by the conjunctive neurons in the second layer (L2) of Fig. 1.

Such a conjunction of activity patterns has been considered important for meaningful information processing, efficient storage, retrieval from partial cues, pattern completion and generalization behaviors in the brain^2^. Conjunctive neurons allow for hierarchical encoding of information by spatio-temporal convergence of inputs which show up as “Trial Type Cells”^3^. Conjunctive binding is a key operation in mechanistic models for hippocampus^2^, brain inspired computational models^4^ and VLSI architectures^5,6^. Rats develop a conjunctive code for context and order^7^ and for different objects within a visual scene^8^. Recordings of single hippocampal neurons show that encoding is coarse-conjunctive^9^. Multiunit activity could be grouped according to behavioral task in progress^10^. Cells in EC conjunctively encode position and head position information^11^. Different face features decoded from single neuron recordings in IT shows coarse tuning of neurons^9^. Firing of hippocampal cells which encode spatial map also correlate to task events^10^. It is also suitable for function approximation and generalization by an artificial neural network^12^.

Modeling studies suggest that combination of features in the stimulus input can be distinguished by a distribution of activation of many neurons. It is also conceivable that the output from many coarse conjunctive neurons converge to one or few ‘output’ neurons that in turn control behavior. In the mushroom body of the common fruitfly Drosophila melanogaster, structural layers of the kind illustrated in Fig. 1 exist. Output from ~2000 third order kenyon cells that encode odour stimuli, converge on ~21 structurally distinct olfactory bulb output neurons (OBONs)^13^ and a suppression of a single pair of OBON regulates aversive memory associations^14^. However, the way information is encoded and decoded across different layers before it converges on the output neuron is not known.

Neuronal cultures on multi-electrode arrays have been previously used to study neuronal networks. The ability to train neuronal cultures has been studied^15^. Different groups have used such cultures to demonstrate processing of spatio-temporal stimuli^16–23^. They have been used as a model to study the network basis of neurological disorders and recently to study the role of neurotransmitters in neuronal network dynamics^24–26^. Their activity has been modeled using connectivity maps and hidden markov models^27^. They have been used to construct simple computational systems. However, such systems have not been used to test different hypotheses about the network architectures for computing using neuronal circuits.

In this study, we have attempted to understand how coarse encoding arises and how features related to the input are encoded by a distributed system of neurons connected randomly using neurons cultured on multielectrode arrays. First, we show that responses from a neuronal ensemble grown on multi-electrode array show coarse-conjunctive encoding of multiple spatio-temporal inputs and then demonstrate their ability to do context dependent encoding, which can then be decoded/classified robustly using ‘perceptrons’ as proxy for the output neuron shown in layer 3 (L3) of Fig. 1. The inputs are paired electrical stimuli at different spatial locations, in different combinations separated by a time interval (spatio-temporal pattern), whose physical parameters were fixed much like the sensory stimuli in the cognition experiments where the perception of sensory stimuli with fixed physical features are studied for context dependency. The results show that neuronal ensembles with probabilistic ‘random’ connectivity can inherently do coarse-conjunctive encoding, without any specific learning or training. We discuss the relevance of such an architecture, where an interplay of random connectivity and layered architecture simplifies the pattern classification tasks.

## Methods

### Neuronal Culture

All animal experiments were performed in accordance with guidelines, rules and regulations of the Institutional Animal Ethics Committee (IAEC) for animal experiments of the Indian Institute of Science, Bangalore, India constituted as per article number 13 of the CPCSEA (Committee for the purpose of Control and Supervision of Experiments on Animals, http://cpcsea.nic.in) rules, laid down by Government of India.

Neuronal culture growth and maintenance was using standard procedures^22,28^. Briefly, dissociated neuronal cell cultures were prepared from hippocampus of 0–2 day old wistar rat pups on 120 MEA from MultiChannel Systems. Micro-dissected hippocampus was digested in papain solution and plated on electrode region of the MEA coated with laminin. The dishes were flooded with 1 ml of medium after the cells had adhered to the substrate, and stored with ethylene-propylene membrane lids in a 65% RH incubator at 37 °C, 5% CO_2_.

We used antibiotic/antimycotic drugs to control contamination. Feedings consisted of 50% medium replacement twice per week. The medium was used with glial conditioning (ara-C) after 7 days.

The culture dish was placed in a separate incubator which maintained an ambient of 5% CO_2_ at 37 °C while doing recordings and stimulations.

### Recording and Stimulation

We used MEA-2100 System from MultiChannel Systems©, Germany for recording from and stimulating the cultures grown on the MEA. The hardware was used to record signals from 120 channels simultaneously at 50 kHz and to generate stimulus pulses at all electrodes under software control.

### Analysis

The data was acquired from the device using MATLAB. Spike detection was done on the acquired data for further processing. This required filtering, artifact suppression and appropriate threshold crossing detection which was done on-line using MATLAB. Threshold for each electrode was estimated as 5x standard deviation (estimated using median values) and was applied on the absolute value of the signal.

For electrical stimulation we chose the parameters which have been shown to be effective in previous studies^29^. For each stimulus we used a bi-phasic voltage pulse of amplitude 500 mV and a pulse width of 500 µs in each phase.

### Experimental Protocols

#### Input Patterns

A spatio-temporal input coding strategy was adopted^22^. 8 electrodes which evoked maximum response from the culture on stimulation were selected as input electrodes and were labeled A,B,C,D,E,F,G,H. These gave us spatially distinct sites for stimulation. Spatio-temporal patterns were created by pairing two of these electrodes at a time resulting in 56 different patterns (AB, BA, AC…). The time interval between pulses was set as 0.5 ms and 3 ms. The patterns were applied to the culture with a time interval of 250 ms.

#### Output decoding

We defined the output vector from the culture for each pattern as a 120 element binary vector indicating the occurrence of a spike in a 100 ms post stimulus window.

$${{\rm{X}}}_{{\rm{jk}}}=[{{{\rm{s}}}^{1}}_{{\rm{jk}}},{{{\rm{s}}}^{2}}_{{\rm{jk}},}{{{\rm{s}}}^{3}}_{{\rm{jk}}}\,\_\,\_\,\_\,{{{\rm{s}}}^{120}}_{{\rm{jk}}}]$$

X_jk_ is the output pattern for the culture for the k^th^ presentation of input pattern j. Here s^M^_jk_ is the spike occurrence indicator for electrode M and is defined as s^M^_jk_ = 1 if at least one spike occurs in the time window 5 ms to 100 ms after the j^th^ input pattern is presented to the culture k^th^ time.

A perceptron is a simple processing element which does a weighted sum of its inputs and generates a binary (1/0) output if the sum is greater than a threshold value. It can be described by the following expression.

$$\begin{array}{llll}{{\rm{O}}}_{{\rm{jk}}} & =\, & \,1\, & {{\rm{W}}}_{{\rm{j}}}\ast {{\rm{X}}}_{{\rm{jk}}} > {\rm{Threshold}}\\  &  & 0 & {\rm{otherwise}}\end{array}$$

Here O_jk_ is defined as the output of the perceptron j with a weight vector W_j_ for the k^th^ presentation of input pattern j.

The weight vector describes a hyper plane which separates the set of outputs which the perceptron is trained to identify from the rest. These set of weights are learned using the perceptron training algorithm, the delta rule^30^.

The decoder is an array of such perceptrons which can be used to assign a class to an output vector.

## Results

We stimulated the culture with 56 spatio-temporal input patterns and recorded the responses. These were generated using 8 electrodes (labeled A, B C D..H) Pairing two at a time with a time delay of 0.5 ms^22^. We defined the first electrode to be stimulated to set the ‘context’ in which subsequent stimuli are processed.

**Figure 2:**
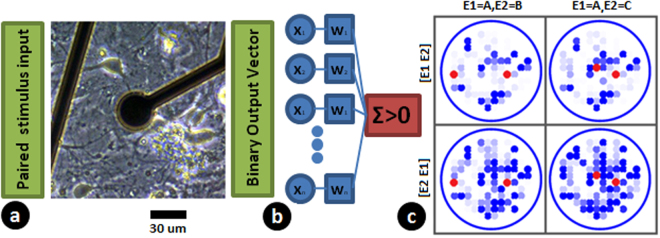
Experimental setup and data. (**a**) Neuronal culture grown on multi-electrode array used for studying conjunctive binding of inputs. (**b**) Responses from the culture converted to binary vectors and perceptron decoders trained to classify patterns. (**c**) Different response patterns on the multi electrode being created when a pair of electrodes (highlighted in red) are stimulated with a delay of 0.5 ms between stimuli. Each blue dot represents an output electrode and the intensity of the dot shows the probability of observing a spike within a 100 ms time window post stimulus. A particular paired stimulus was repeated 45 times to calculate the probability of observing the spike. One can see that the pattern depends on the electrodes being stimulated and order of stimulation.

**Figure 3:**
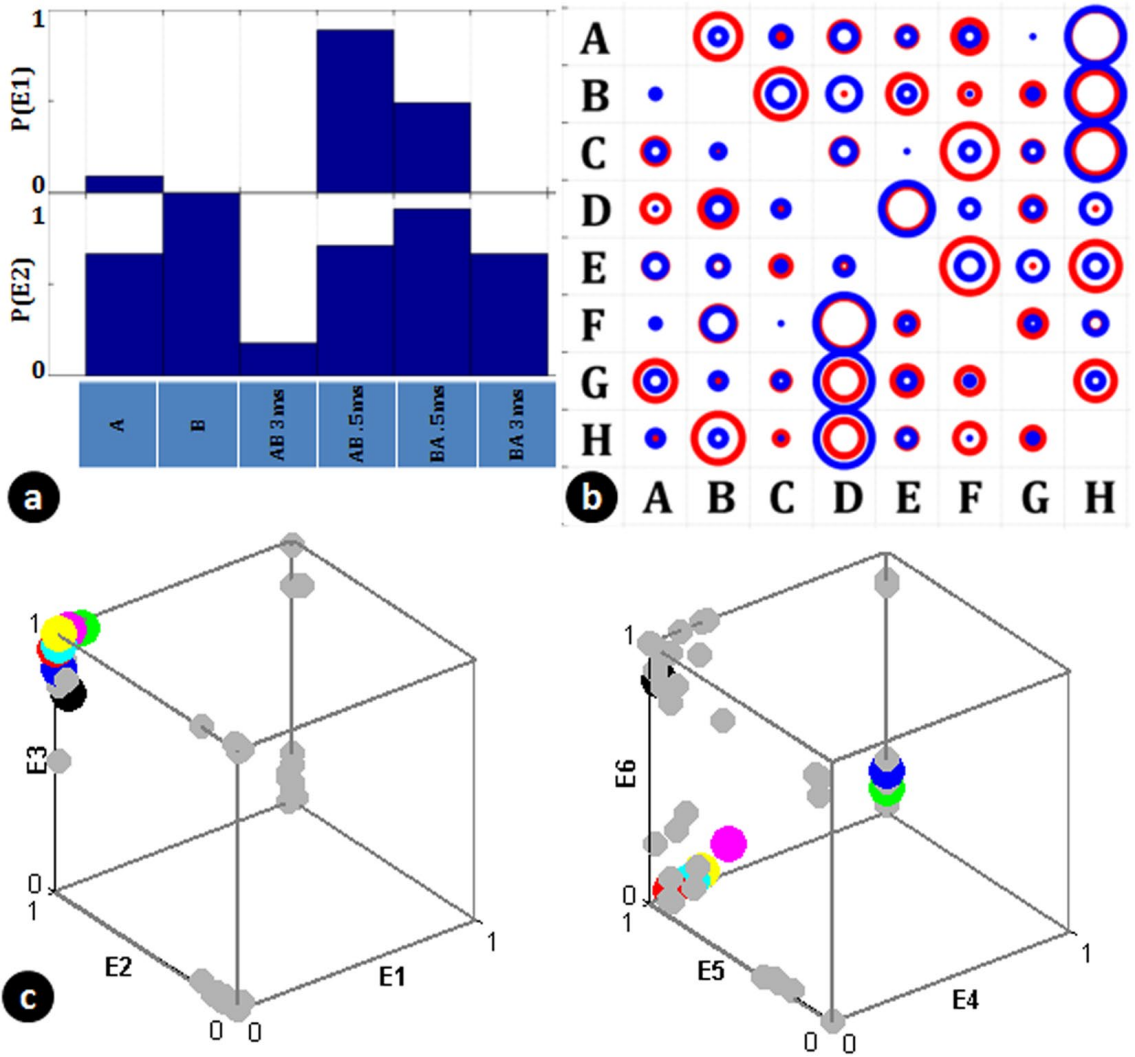
Output of the neuronal culture for different stimulus patterns reveal a coarse coded conjunctive scheme of encoding. The data presented is from a single experimental trial on a neuronal culture. (**a**) Conjunctive Encoding. An electrode (E1) which shows a response only on pairing (maximum response when paired with a time delay of 0.5 ms). Disjunctive Encoding, an electrode (E2) where a response is suppressed on pairing (Silencing on pairing with a time delay of 3ms). Height of bar indicates probability of response (**b**) Two electrodes (Blue and Red used to indicate the electrode) showing a coarse tuning response (relative size of the circle indicates probability of response with response to AH having a probability 1 at both electrodes) to various stimulus patterns. The response is not specific to a particular electrode or a particular pattern. (**c**) Coarse coding generates distinct codes for different patterns. This shows how six electrodes (selected using Fischer Discriminant Ratio) create unique codes for different groups of input patterns. Each dot corresponds to the probability of firing observed at these electrodes for different stimuli. Consider input pattern corresponding to dots RED(R) and GREEN (G). When only electrodes [E1, E2, E3] are used for decoding (LHS), the coordinate generated for R is [0, 1, 1]. This is true for green dot as well and these two patterns cannot be distinguished. However, when [E4, E5, E6] is also used, the combined coordinate([E1, E2, E3, E4, E5, E6]) generated for R will be [0, 1, 1, 0, 1, 0] whereas for G, this is [0, 1, 1,1, 1, 0], which are now linearly separable. Thus with sufficient number of electrodes, unique descriptions/coordinates are created for every pattern. This is illustrated conceptually in Fig. 1(f–h), where unique codes develop when sufficient number of coarse conjunctive neurons are used for decoding.

**Figure 4:**
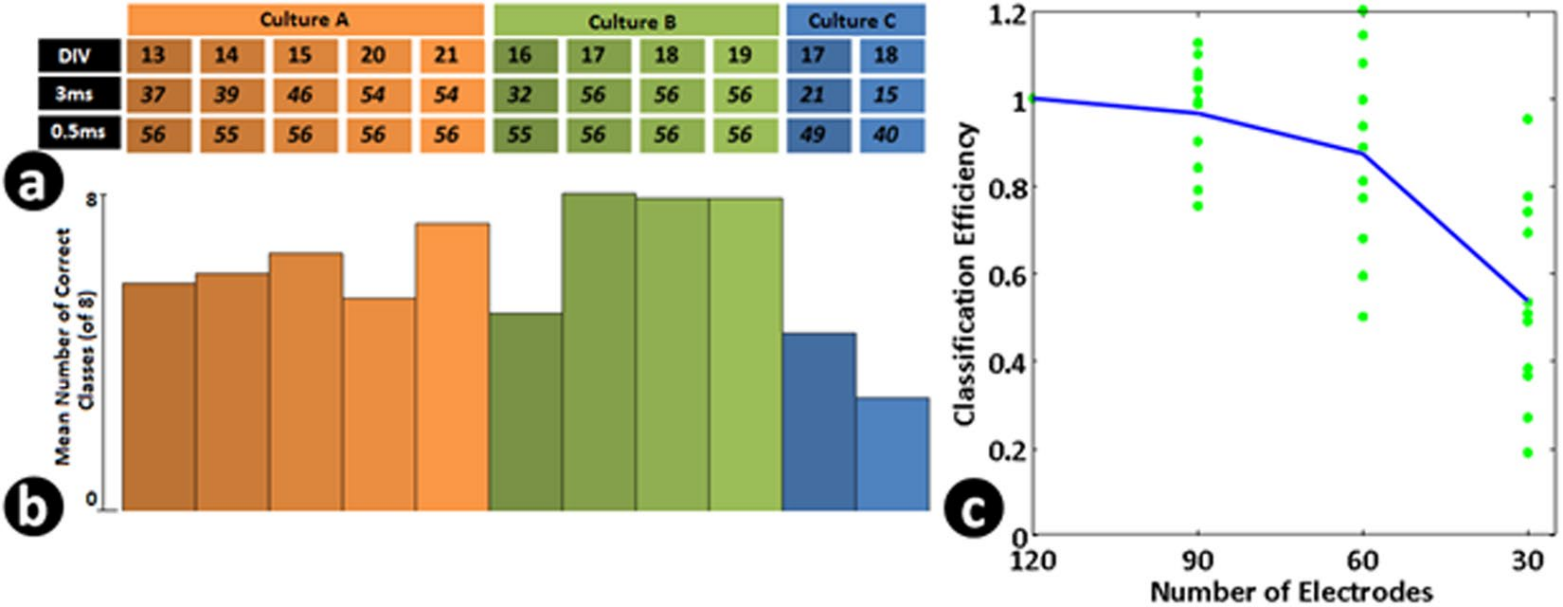
Coarse-Coded Conjunctive scheme results in similar yet distinct representations for related input stimuli. This allows the culture to do pattern generalization and novel pattern classification. Such a scheme is also shown to be robust to probabilistic nature of connections. (**a**) Output Classification accuracy for the 56 paired stimulus patterns over different trials in different cultures (C) studied on different days in culture (DIV, days *in vitro*). The numbers indicate the number of patterns classified with an accuracy greater than 80%. The results are shown separately for a time delay of 3ms and 0.5ms of pairing. (**b**) Classification ability of the cultures for a novel pattern. 8 patterns, one from each group of paired stimuli (indicated for example in Fig. 2 were systematically left out and the perceptrons were trained to classify rest of the inputs into different group. The height of the bar indicates the average number of different such hidden patterns (out of 8) that were correctly classified (with greater that 80% accuracy (Chance = 1/8)). This indicates that patterns are grouped into linearly separable groups in higher dimensions based on the first electrode stimulated. (**c**) The coding of the outputs is such that a perceptron connected probabilistically to a fraction of output electrodes is able to classify the inputs without significant degradation. The curve is averaged over 11 trials in Fig. 4a. It indicates the reduction in number of input classes correctly classified with greater that 80% accuracy as the number of connections each perceptron receives is reduced. The blue trace indicates the loss of accuracy when perceptrons are connected randomly to a fraction of output electrodes (randomized 3 times and mean number of classes calculated). This loss of accuracy can be seen as illustrated in Fig. 1(f–h).

We then looked at responses at each electrode for these patterns and found them to be coarsely tuned with multiple electrodes responding probabilistically to the 56 input patterns (Fig. 2c). With an array of perceptrons, we were able to classify the output codes which showed them to be linearly separable^22^ (Fig. 4a). This method has been shown to be equivalent to other classification methods like logistic regression^31^. Figure 3b shows coarse tuning at two electrodes with responses to multiple input patterns. The output response from a single electrode (responses represented by blue dots in Fig. (3b)) cannot distinguish the different input patterns (DF, DG, DH, HA, HB, HC). The probability of the responses at a single electrode show conjunctive and disjunctive behavior based on timing and order of inputs as a result of excitatory and inhibitory connections from the inputs (Fig. 3a). Looking at all the electrodes, we found that a significant number of electrodes show this kind of response leading to distinct population codes (inferred based on them being linearly classifiable by perceptrons). The input patterns become distinguishable as small clusters upon increasing the number of output electrodes (as in example demonstrated using Fig. 3c). A minimum number of output electrodes are thus necessary to separate the input patterns. With 120 electrodes the input patterns were separable and classifiable. Thus coarse-conjunctive coding results in unique population codes.

The paired input patterns could be grouped into 8 sets ([AB, AC…], [BA, BC…]). 8 linear perceptrons were trained to classify them successfully showing the grouping of output responses based on first electrode stimulated in the sequence.

In order to study context dependent grouping of the inputs, we left out one of the patterns within each group where the first stimulus sets the context (e.g. AH in group [AB, AC, AD, AE, AF, AG, AH]) and trained the output perceptrons.

To be able to identify the group of input, we had 8 perceptrons. Each perceptron was trained to respond to a presentation of a pattern belonging to a particular input group. When the training was done, one set of patterns was randomly left out (say [AH, BH, CH, DH, EH, FH, GH]). After training, to check the ability of the perceptron to identify a novel input pattern from the network response, we presented the pattern (say AH) to all the perceptrons and evaluated their response. Then using a winner-take-all strategy, the pattern was assigned to the group corresponding to the perceptron that shows highest activation (In the example, this should be A*). This was repeated for other left out patterns (BH, CH etc, 45 samples each) and the classification accuracy for each of these were noted. If 80% of the samples of a pattern were correctly grouped, we say that the perceptron layer was able to identify the novel group correctly. The number of such groups was noted. This was repeated by leaving out other set of patterns (say [AG, BG, CG…], [AF, BF, CF…]) and similar analysis was performed. Figure 4b presents an average number of correct groups thus identified by each culture.

The fact that the output generated by a pattern AH was grouped into [A*] group instead of [H*] group indicates that the network response is strongly influenced by the first stimulus in the sequence rather than a co-occurrence of A & H. Together with the results that each pattern is distinct (56 patterns were linearly separable and patterns within each group were linearly separable) but also can be grouped (A*, B*..) while being able to classify a novel pattern, shows the ability of coarse-coded conjunctive scheme in neuronal cultures to create unique descriptions suitable for pattern classification and pattern generalization. The ability of the perceptron to do this, shows that the network dynamics and resulting response is such that a neuron in the next layer is able to group inputs correctly.The results on the ability to correctly classify untrained patterns emphasizes that the classification ability is not just due to a mapping to high dimension and demonstrates a ‘context’ dependent response to the second input and shows the inherent network property to generate such responses.

To check if the coding is suitable for probabilistic connections between layers as in the brain, we made the connections between the output electrodes and decoder perceptrons probabilistic and evaluated the classification performance. We mimicked the possible connectivity in a neuronal architecture by randomly connecting a perceptron in the output layer to a fraction of output electrodes (Fig. 1). The performance was robust and degraded gracefully as number of connections were reduced (Fig. 4c). The result indicates that the code generated by coarse-conjunctive neurons is distributed enough to allow a neuron randomly connected to a set of neurons in this layer to learn an arbitrary linearly dependent function.

We created a random network model for a mechanistic description of the stimulus responses for spatio-temporal input patterns from neuronal cultures. We viewed the network as a two layer network with an input layer consisting of stimulated neurons and output layer consisting of neurons directly connected to input neurons. This allows us to view cultured network as a layered architecture. Such network structures are used in studying computational capabilities of neuronal systems, brain-inspired computational frameworks and artificial neural networks. We studied how our experimental setup could mimic these computational models. The membrane and synaptic time constants were constrained biologically. Crucially, this points to the possibility of studying computational properties and learning capabilities of biological layered networks using cultures grown on multi-electrode arrays and investigate if computations done using artificial neuronal networks and brain-inspired frameworks can be done using biological systems.

**Figure 5:**
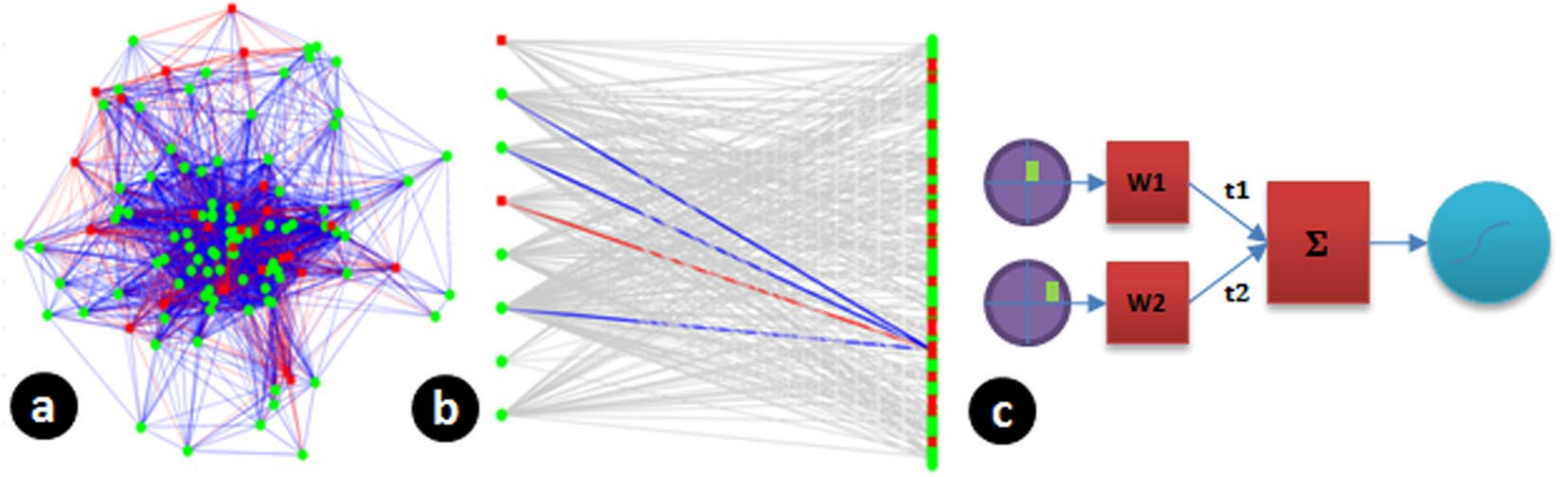
Modeling a random neuronal culture to analyze first spike response to a spatio-temporal stimulus pattern. (**a**) A neuronal network generated with random locations of neurons and distance dependent connection probabilities. Green represent excitatory neurons and red show inhibitory neurons (**b**) The network viewed as a two layer network after selecting 8 neurons as inputs to analyze first spike response behavior. Connections from input electrodes to a single output neuron is highlighted. Such a partial connectivity is hypothesized to give rise to a coarse-conjunctive population code at the output layer. (**c**) Model assumed for calculating output firing probability for a paired stimulus at an output electrode. Inputs are stimulated with a delay of t_d_, a weighted sum is calculated to determine excitation at an output electrode and a sigmoid function is used to calculate output firing probability.

The model as shown in Fig. 5, had 120 neurons, with 80% being excitatory and the rest inhibitory. Each neuron was supposed to mimic an electrode and we expected this two layered architecture to explain the observed spiking probability. The probability of firing of a neuron was calculated as a sigmoid function of weighted sum of inputs coming into the neurons.

The connectivity between the neurons in the model was set using two methods.

In the first method, we had a global parameter ***p*** which defines probability of connection between any two neurons in the network. We tuned this parameter so that when the input patterns are applied, the output generated by the network has similar overall properties like being linearly separable, sequence dependence and grouping when paired stimuli are applied. We then analyzed the network generated this way for a number of connections received by each neuron to allow it to mimic the observed behavior of the biological network. This provided further validation of schema for computation using layered architectures with random connectivity.

In the next method, we estimated the functional connectivity and the connection weights between the input and output electrodes in the neuronal culture by fitting the model outputs for different paired stimuli to match the probability of firing of output electrodes in experiments using a combination of genetic algorithms and gradient descent. The genetic algorithm tuned whether or not a connection exists between neurons while the gradient descent tuned the connection weights. Using this approach, we had a network which had firing probabilities at different electrodes close to the experimental data. The validity of the model-fit was established by using the model to generate output vectors and analyzing them in the same way as experimental data. We then compared the connectivity in this network with that of the network generated by the first method to see whether the number of connections are similar. In the first method, the connection probability is used to manipulate the connectivity, while in the second method the experimental data is used to do so. Since both methods can now recreate the overall experimental results in simulation, we were more confident of the model network explaining the observed behavior and use connection probability as a parameter to further study how connection probability might affect network performance.

**Figure 6:**
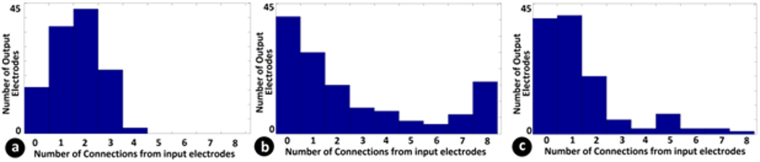
Connectivity estimates between input and output electrodes: (**a**) Histogram of number of input electrodes connected to an output electrode in a model generated with random connectivity to replicate the features of the experimental data. (**b**) Connectivity estimated from the experimental data by fitting the model where experimental data has a stimulus pairing interval of 0.5 ms and (**c**) with 3 ms. It can be seen that connectivity profiles look similar with an output electrode receiving connections from 3 input electrodes. Also more connectivity is seen with a 0.5 ms pairing showing that temporal integration is more effective at this level of pairing.

Using the functional connectivity so obtained, we got further insights about the structure of the network. Figure 6 shows a histogram of number connections between input and output electrodes for a randomly generated connectivity between electrodes and those estimated using fitting the model to the data. They are in agreement to an extent that on an average, an output neuron has a functional interaction with 3 input electrodes for the network in the culture. Also, the higher number of functional connections estimated when the delay between pairing is 0.5 ms indicates that for these networks the dominant cause for generating conjunctive neurons would be through overlap of EPSP’s from multiple inputs.

**Figure 7:**
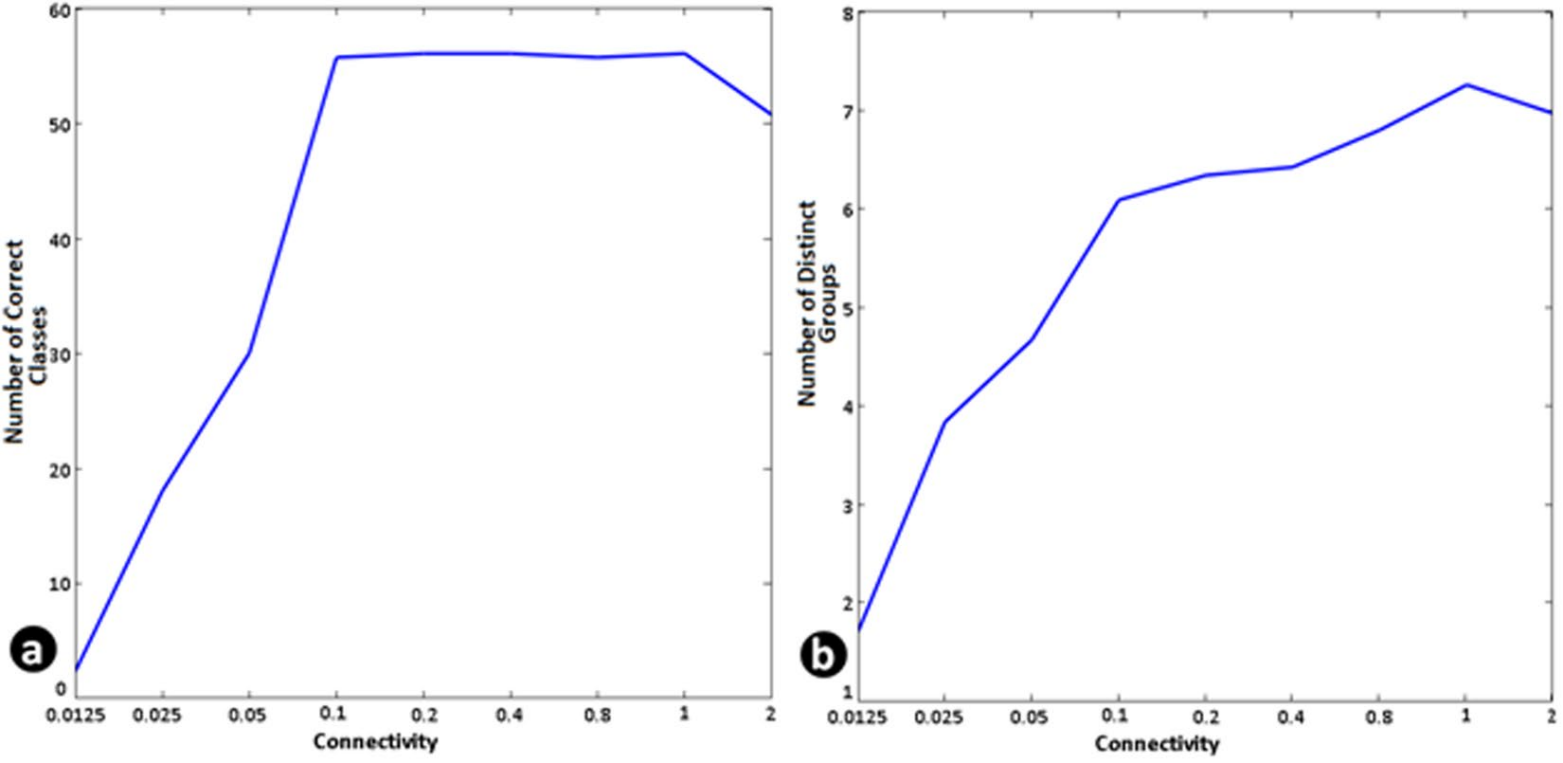
Classification accuracy depends on sufficient connectivity and conjunctive neurons in the network. (**a**) Number of distinguishable classes (of 56) and (**b**) Number of groups which show correct novel pattern identification when degree of connectivity in the network is changed. It can be seen that with low connectivity resulting in less number of conjunctive neurons the classification behavior is not observed. These results are in agreement with data presented in Fig. 4.

We then varied the number of connections between the neurons to see how it affects the classification performance. This is shown in Fig. 7 where the connectivity parameter (p) is varied and the classification and grouping abilities of the model network is studied in the same way as with the biological network. As expected, we found that a minimum degree of random connectivity is required for generating sufficient number of coarse-conjunctive neurons. Interestingly, with the parameter value at 0.1 where the model shows a 100% classification ability for 56 classes, the number of distinct groups possible was around 6 which was similar to the observation across multiple neuronal cultures as presented in Fig. 4.

## Discussion

We discuss the relevance of the above findings in the context of computing mechanisms in the brain. Currently, it is not clear how the functional connectivity in the brain changes and to what degree, in order to learn to perform some action. Also learning of a precise weight at different layers in a neuronal network would be a difficult challenge without accurate feedback signals and would require many repetitions of the training as experienced by researchers working with deep learning networks. It is also not clear how the equivalent error correcting mechanisms would work in a biological neuronal network.

In the following discussion, we show that with an interplay of random connectivity and a layered structure, neuronal circuits can solve such problems without needing to learn a large number of synaptic weights. We show how our experiments and modeling studies support this hypothesis for neurons cultured on multi-electrode arrays.

### Linear separability as a key intermediate step for problem solving

Identification of the correct features from the data and transforming the inputs to a linearly separable space has been established as a key intermediate step in problem solving in machine learning. The ‘kernel’ in the support vector machine based classification, ‘hidden layer’ in artificial neural networks, the ‘random network’ in LSM’s, all use this same principle. Once the problem has been thus translated, the required arbitrary function to be learned is a linear combination of these outputs by a single neuron obtained by tuning its input synaptic weights, without needing large scale modification of the preceding input network (Fig. 1). Such learning of a linear combination of inputs, has been shown to be theoretically possible for a biological neuron equipped with STDP mechanism^32^. Specifically, classification and pattern recognition can be seen as a special case of thresholding of these linearly combined outputs. In our study, using 56 input patterns we have shown that the output of the neuronal culture shows such a transformation property (Fig. 4a). The output of the culture, which encodes the input stimulus into a higher dimensional representation, are linearly separable via perceptrons, and learn functions like classification, grouping, sequence detection and novel pattern recognition (Fig. 4b). Previously we have shown that such a biological neuronal network in culture on multi-electrode arrays can translate linearly un-separable inputs to a high dimensional linearly separable space^22^.

### Conjunctive neurons create linearly separable population codes

The generation of linearly separable population codes can be explained using the schema for a hypothetical network shown in Fig. 1. Experimentally, we show that each neuron in the randomly interconnected network shows a conjunctive code (Fig. 3b). This most likely arises out of pairing of excitatory and inhibitory pre-synaptic inputs when two electrodes in the array are stimulated within a time window (Fig. 3a). Both excitatory and inhibitory connections are required for the neurons to show both an increase and decrease in firing probability as a result of pairing. Such connections also allow the neurons to detect the order of firing. Our results on the neuronal culture show that single neurons receiving random connections show ‘conjunctive encoding’ which are sensitive to electrodes being stimulated, their timing and the order of pairing, akin to the ‘conjunctive neurons’ demonstrated *in vivo*. The additional observation of ‘disjunctive encoding’ suggests the presence of both excitation and inhibition and their importance in the generation of a variety of conjunctive neurons with arbitrary inputs (Fig. 3a). A linearly separable population code can emerge from a sufficient collection of such randomly connected neurons. This finding emerges from our analysis of the output data, by using random subsets of output electrode data for classification (Fig. 4). These results emphasize the importance and sufficiency of randomly connected neurons to create such population codes without needing any specific learning/training of these networks (Fig. 7).

### Neurons show coarse conjunctive coding

The results shown in Fig. 3b indicate that single neurons can show a coarse conjunctive response, i.e., each neuron is responsive to pairing of multiple spatio-temporal inputs. The presence of coarse-conjunctive neurons has been shown in the mammalian brain and its importance and advantages have been highlighted in theoretical studies^2^ . Coarse-conjunctive codes makes the encoding of the inputs robust as schematically illustrated for hypothetical network in Fig. 1. With such a code, a larger number of patterns can be represented by the network without needing a conjunctive neuron for every feature in the input space (Fig. 1). A decoding neuron in the final layer (L3), only partially connected to such a population of coarse encoding neurons from preceding layers, can still have sufficient information for decoding. Our analysis with random connectivity between the neuronal culture and output layer perceptron demonstrates this to be true for neuronal cultures on MEA (Fig. 4c). Such a scheme is suitable for structured yet probabilistic connections as found in biological neuronal systems.

### Coarse conjunctive encoding emerges out of random connections without specific learning

Distinct coarse-conjunctive neurons can emerge out of random connectivity between two layers in a network. Our modeling study inspired by our experimental data shows this to be true (Figs 5 and 6). Our analysis with the model also shows that a minimal connectivity is required for generation of such a code (Fig. 7). Conjunctivity arises due to firing of inputs within a time window and depends on the electrodes from which it receives connections, which can be random. The ability to detect the timing and order of firing depends on the inherent time delays in the circuit and the presence of excitatory and inhibitory connections. Neurons show coarse-conjunctive encoding as it receives inputs from more than two electrodes. Each neuron has a distinct coarse-conjunctive tuning curve due to the random nature of connections. A sufficient number of such connections create a set of neurons which can project the inputs into a high-dimensional linearly separable space.

### The robust nature of the encoding allows the subsequent layer of neurons with partial connectivity to learn an arbitrary function

An intermediate layer receiving random connectivity from a previous layer generates a robust encoding using coarse conjunctive neurons. As a result of such a code, a perceptron, probabilistically connected to this layer is able to identify the input pattern or a group of inputs. By extension, a neuron equipped with STDP should be able to achieve the same. Significantly, to learn a new class, instead of a large-scale change to all the synaptic weights in the network, only the weights of a single target output neuron connected randomly to the preceding coarse conjunctive neurons, needs to be modified.

In conclusion, we have shown that random neuronal networks in a culture, generate coarse-conjunctive outputs and unique population codes that are linearly separable for different input sequences without any specific training of the culture. The findings have physiological relevance in giving us some preliminary understanding of how neuronal networks in the brain might sift through information and implicitly classify them intrinsically, via linearly separable, highly redundant, coarse conjunctive encodings of the input stimulus, without needing explicit training/learning at all functional layers during information flow. Such an encoding ability might have a great utilitarian role in simplifying the learning process by needing modification of only a few final neuronal layers, as opposed to the entire network. However, this conjecture requires further experimental analysis of neuronal recordings from the brain *in vivo*.
